# A novel flow-based geometrical upscaling method to represent three-dimensional complex sub-seismic fault zone structures into a dynamic reservoir model

**DOI:** 10.1038/s41598-019-41723-y

**Published:** 2019-03-28

**Authors:** Md Saiful Islam, Tom Manzocchi

**Affiliations:** 1 Fault Analysis Group, Science Centre West, UCD School of Earth Sciences, University College Dublin, Dublin, Ireland; 2 Department of Mechanical and Mechatronics Engineering, College of Engineering, Dhofar University, Salalah, Oman

## Abstract

Most hydrocarbon reservoirs contain faults, which are highly complex heterogeneous and anisotropic three-dimensional (3D) volumes of deformed rock. A major technical challenge in full-field flow simulation is to represent the effects of 3D fault zone structure within the two-dimensional (2D) fault planar surfaces using the industry standard commercial reservoir flow simulator due to its limited functionality. Therefore, a new flow-based geometrical upscaling (FBGU) method has been developed for capturing the effects of 3D fault zone structures in conventional low-resolution upscaled flow simulation models. Geometrical upscaling (GU) is the process of calculating across-fault and up-fault transmissibility arising from 3D flow paths through fault zones, and expressing these transmissibilities as implicit connections in a low-resolution upscaled flow simulation model. The high accuracy of the method is demonstrated by comparing the flow responses of high-resolution (referred as truth model in this paper) simulation models in which the 3D fault zone structure is represented explicitly in the grid geometry, with that of conventional resolution models in which it is upscaled using FBGU method. The flow results show that the newly developed FBGU method is extremely accurate and geometrically flexible.

## Introduction

In many hydrocarbon reservoir management studies, detailed geological models with different scales of heterogeneities are generated. Upscaled versions of these models are routinely used in flow simulation history matching studies in order to predict future behaviour of the reservoir and to optimize development decisions. In the geological modelling process, several scales of heterogeneities, representing both petrophysical properties (e.g. permeability, porosity or net-to-gross variability) and geometrical complexity (e.g. faults, facies transitions, pinch outs) are encountered and, if important to flow performance, all of these heterogeneities need to be preserved in the upscaled numerical flow simulation model. Commercial numerical flow simulators commonly can deal with approximately 10^4^ to 10^5^ cells, and the precise number of cells may vary depending on the available computer memory and complexity of the reservoir modelling study. However, a typical 3D fine-scale geological model contains details generated stochastically and may comprise 10^6^ to 10^9^ or more cells^1,2^. Therefore, upscaling is needed to redefine the high-resolution geological model to a low-resolution upscaled flow model. This scale-up must preserve the effects of heterogeneities on the overall flow in the numerical flow simulation models.

Many researchers have investigated and developed single-phase (i.e. absolute permeability, porosity, net-to-gross ratio) and two-phase (i.e. pseudo relative permeability and pseudo capillary pressure) upscaling procedures^1,3–8^ for dealing with small-scale volume-filling petrophysical variability. Small-scale geometrical features which occupy only a small fraction of the volume they are contained in, such as sub-seismic faults or shale-draped sedimentary structures, have received less attention and if they are considered in upscaling studies it is generally only with reference to establishing effective permeabilities due to low permeability fault rock or shale drapes^9–11^. However, low permeability fault rock is not the only characteristic of faults to consider, and shear displacements across sub-seismic faults can be very significant on reservoir flow. Effects of uncertainties in across-fault juxtapositions arising from uncertain fault displacement are much more difficult to predict and manage than effects of uncertain fault rock^12–14^. Methods for sub-seismic fault prediction^15–17^ are beyond the scope of the present study, which instead focuses on the modelling of it.

Manzocchi *et al*.^18^, first introduced geometrical upscaling (GU) specially for capturing the juxtaposition effects of sub-seismic fault zone structures into field-scale flow simulation models and this method is referred as template-based geometrical upscaling (TBGU) method. Nonetheless, some other researchers have modelled small-scale sub-seismic fault zone structure using high-resolution flow models by local grid refinement (LGR)^19–24^. For example, Havana^22^ is such a fault-modelling tool using LGR, which has more recently been applied in the Fault Facies (FF) research program. The LGR technique aims to capture the influences of fault zones with heterogeneous petrophysical properties into flow simulation models as deformed rock volumes. In this method, fault zones are isolated from the coarse grid model and split into high-resolution refined grids, which are then merged in the coarse grid model at the equivalent location. The GU method allows fault zone structure to be represented in conventional low-resolution flow simulation models without changing the geometry of the flow simulation model, or requiring a locally-refined grid. Instead, neighbour and non-neighbour connections representing flow paths within, across and along the segmented fault are calculated as a function of transmissibilities and output at the resolution of the full-field model. In the TBGU method^18^, the connection transmissibilities are determined using a three-dimensional template which can be modified to reflect the geometry of intact or variably breached and variably rotated relay zones and other types of fault lens. This TBGU method has been applied to test the sensitivity of full-field reservoir production behaviour to a range of fault-zone related uncertainties including the presence of realistic densities of relay zones, damage zones and paired slip surfaces, with results indicating a reservoir-specific dependence on the importance of fault segmentation on reservoir flow^18^. Downsides of the TBGU method are that the template is not sufficiently flexible to include all possible forms of sub-seismic fault zone geometry, and that the upscaled flow path transmissibilities are not particularly accurate^25,26^. The objective of this paper, therefore, is to introduce a new flow-based geometrical upscaling (FBGU) approach, which is both more accurate and more flexible than the existing TBGU approach and the LGR method. The modelling workflow of the new FBGU method is presented in the following section. The existing TBGU method is described in the supplementary information section to understand the study and to justify the development of the new FBGU method (please see the Supplementary Notes 1, 2 and Supplementary Figs S1 and S2 for the existing TBGU method and its limitations).

### Modelling workflow of FBGU approach

**Figure 1:**
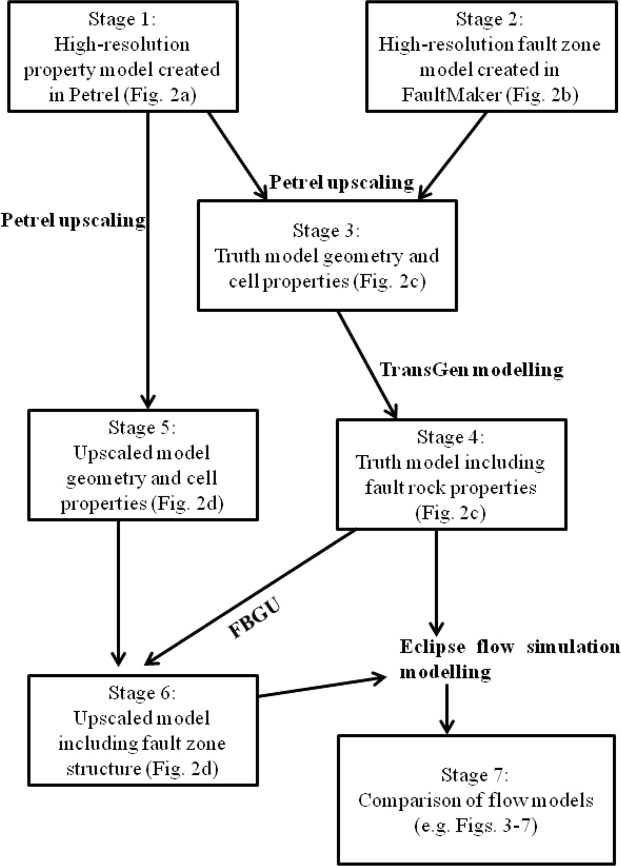
Flow chart of the stages of models applied in this study. Arrows represent modelling processes and the software tools used to perform the modelling. The figures cited (mainly in Fig. 2) show stages of the model for Reservoir A.

**Figure 2:**
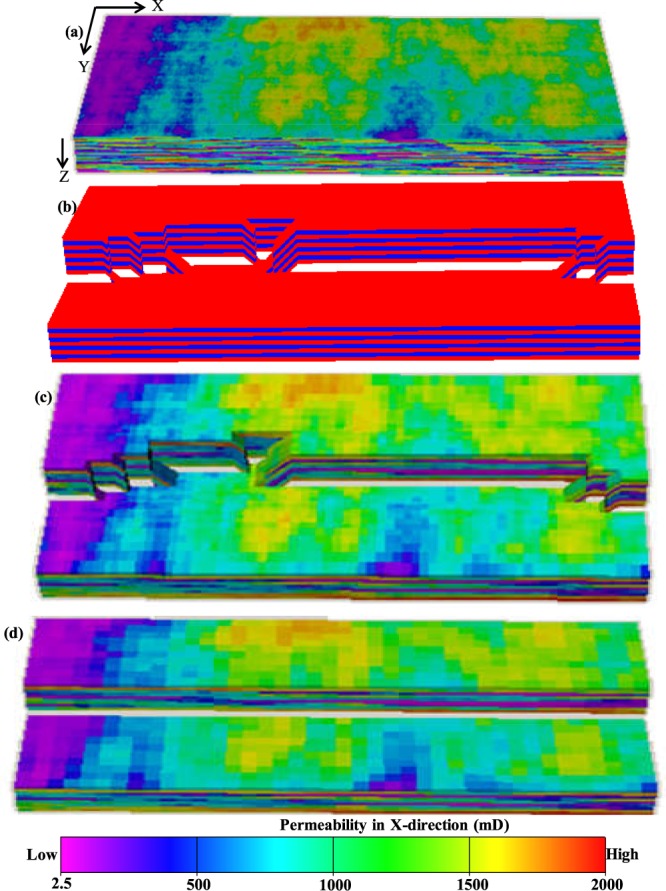
Reservoir model, A at different stages in the workflow. (**a**) Permeability field of a fine-scale geological model generated in Petrel^27^. (**b**) The FaultMaker model that defines the high-resolution explicit fault zone geometry. (**c**) Petrophysical properties (e.g. permeability) are upscaled to the resolution of the FaultMaker model to define the truth model. (**d**) The upscaled property model. The objective is to upscale the fault zones from the truth model into this geometrical framework.

This section provides an overview of the modelling workflow applied in this study to take into account the scale of heterogeneities caused by petrophysical properties and the heterogeneities caused by geometrical complexities such as faults into upscaled flow simulation models. Three different test cases (Table 1) are considered, and all are modelled using the same basic workflow (Figs 1 and 2). The petrophysical properties, sub-seismic fault zone structures and reservoir production boundary conditions are different for each case, so between them the three cases provide a more comprehensive test of FBGU than any one of them alone.

**Table 1:** Dimension, resolution and structures of the models.

Fine-scale geological model	Petrophysical properties are upscaled from fine-scale geological model	
	Truth model	Upscaled model
• Reservoir structures are homogeneous (e.g. unfaulted model)	• Contains 3D sub-seismic fault zone components explicitly	• Contains 3D sub-seismic fault zone components implicitly
• Uniform Grid cells	• Non-uniform grid cells	• Uniform grid cells
• Test model **A**:	• Test model **A**:	• Test model **A**:
° Dimension: 1 km × 500 m × 90 m	° Dimension: 1 km × 500 m × 90 m	° Dimension: 1 km × 500 m × 90 m
° Resolution: 1000 × 500 × 90	° Resolution: 82 × 67 × 9	° Resolution: 40 × 20 × 9
• Test model **B**:	• Test model **B**:	• Test model **B**:
° Dimension: 1 km × 500 m × 90 m	° Dimension: 1 km × 500 m × 90 m	° Dimension: 1 km × 500 m × 90 m
° Resolution: 1000 × 500 × 90	° Resolution: 80 × 52 × 9	° Resolution: 40 × 20 × 9
• Test model **C**:	• Test model **C**:	• Test model **C**:
° Dimension: 2 km × 1 km × 90 m	° Dimension: 2 km × 1 km × 90 m	° Dimension: 2 km × 1 km × 90 m
° Resolution: 1000 × 500 × 90	° Resolution: 84 × 56 × 9	° Resolution: 40 × 20 × 9

The fault zones are embedded in a spatially variable reservoir sequence, and the starting points of the modelling are a high-resolution geostatistical property model (Fig. 2a) and a high-resolution segmented fault zone model (Fig. 2b). The final objective of the modelling is to create a high-resolution flow simulation model in which fault zone complexity is included explicitly (Fig. 2c), and a lower-resolution model in which the fault zone is upscaled and represented implicitly as neighbour and non-neighbour connection transmissibilities (Fig. 2d). This section describes the seven stages applied to generate these models.

In Stage 1, a high-resolution property model is constructed in a regular unfaulted grid (Fig. 2a). This model deliberately has much smaller grid cells than all subsequent models, allowing it to be upscaled to their resolutions in later modelling stages (see below). Properties (porosity, net-to-gross ratio and directional permeabilities) were generated stochastically using the Sequential Gaussian Simulation tool in Petrel^27^, with normal distributions and exponential variograms for each property (Supplementary Tables S1 and S2).

The second input is a high-resolution fault zone model (Stage 2, Fig. 2b). This model was created using FaultMaker, a standalone software allowing the generation of geocellular models of realistically heterogeneous segmented fault zones^28^. In the FaultMaker approach, fault zone components such as intact and breached relay zones are modelled stochastically using similar parameters to those discussed by Manzocchi *et al*.^18^ to determine the frequency and geometry of each component. In the present study, constant throw faults are modelled with between 5 and 10 relay zones along their length.

FaultMaker creates pillar grids with constant layer thickness but variable cell sizes in the two horizontal directions. In Stage 3 of the modelling, the high-resolution regular gridded petrophyscial model is upscaled to coarser, irregular FaultMaker grid. The upscaling was performed using Petrel^27^. The additive petrophysical properties (e.g. porosity and net-to-gross) are upscaled by volume weighted arithmetic average and the non-additive directional permeability is upscaled using the flow-based property upscaling method available in Petrel^27^. The resultant model (Fig. 2c) contains both a heterogeneous sequence and a heterogeneous fault, but is not yet the truth model as fault rock properties have not yet been added.

The truth model is created in Stage 4, by adding fault rock properties to the petrophysical model upscaled to the resolution of the FaultMaker model (Fig. 2c). Single-phase fault rock properties are represented as fault transmissibility multipliers^29^ using a constant absolute fault rock permeability of 0.001 mD, and a fault rock thickness estimated from the correlation with the local fault throw using a ratio of 170 between throw and thickness (i.e. a 100 m throw fault has a 59 cm thick low permeability fault core). The transmissibility multipliers used to assign these properties were calculated in TransGen^30^. In this study, only single-phase fault rock properties are considered, however two-phase fault rock properties (i.e. relative permeability and capillary pressure curves of the fault rock) can also be dealt with during the geometrical upscaling process^31^.

Stage 5 of the modelling concerns the creation of the upscaled grid which is designed to be representative of a portion of a conventional resolution flow simulation model in which faults are represented as offsets between cells (Fig. 2d). The model contains a single continuous fault and a regular grid. Cell properties were upscaled from the high-resolution property model using the same procedures as used in Stage 3.

Stage 6 of the modelling is the principal objective of this study. The structurally complex 3D fault zone model (Fig. 2c) is upscaled to the resolution of the coarse model (Fig. 2d) using the newly developed FBGU approach. Flow paths that exist explicitly in the geometry of the truth model (Fig. 2c) are included as neighbour and non-neighbour connections between cells in the upscaled model (Fig. 2d). In the newly developed FBGU method, incompressible steady state numerical flow simulation is conducted using Eclipse^32^ to calculate the transmissibilities of these connections using Darcy’s law. The working principle and details of FBGU approach is described in the Method Section. In the low-resolution upscaled model (Fig. 2d), the single-phase fault rock properties are incorporated implicitly as their effects are included with the geometrical effects of the fault zones during the upscaling process.

The final modelling Stage (Stage 7, Fig. 1) involves testing the accuracy of the FBGU method. In this stage, the truth and upscaled models are subjected to identical two-phase (i.e. oil is displaced by water) numerical flow simulation tests, and the model behaviours are compared. The summary of the test models used to examine the FBGU method is presented in the Supplementary Information Section (please see Supplementary Note 3, and Supplementary Figs S3–S5 and Supplementary Table S3) and results are discussed in the next Section. Models considered in this study are built exclusively in corner-point geometry (CPG) which is still the most common means of building full-field faulted flow simulation models of hydrocarbon reservoirs^33–39^.

## Results and Discussion

**Figure 3:**
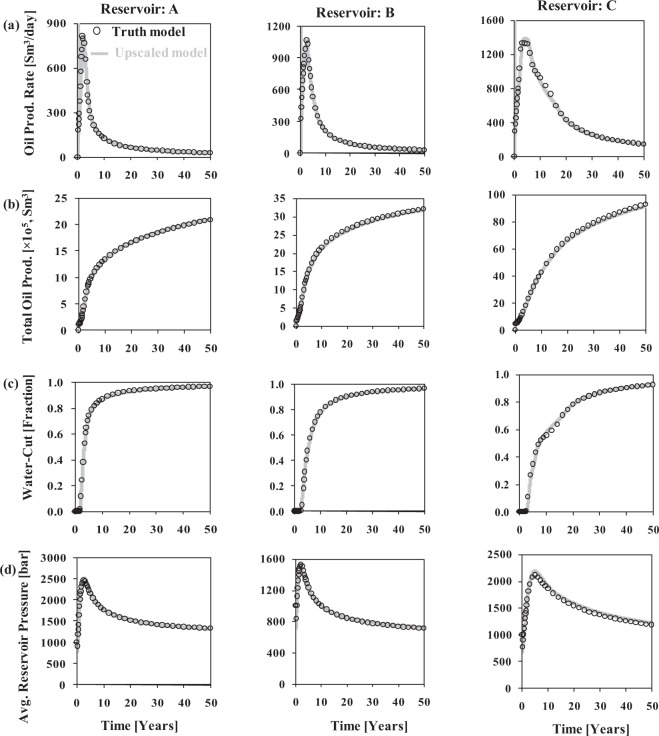
Field wide behaviour of the three reservoirs. (**a**) oil production rate (Sm^3^/day), (**b**) total oil production (Sm^3^), (**c**) water-cut (fraction), and (**d**) average reservoir pressure (bar) over time for the truth model (black coloured circles) and upscaled model (grey coloured solid line).

**Figure 4:**
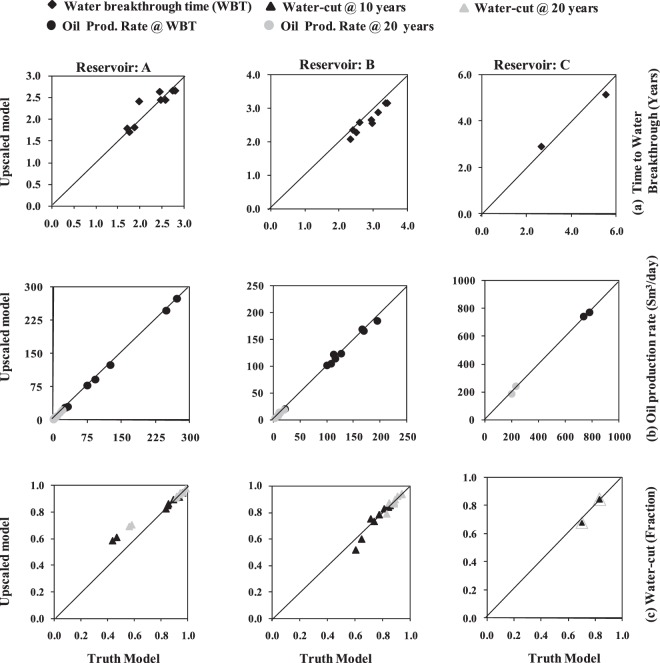
Cross plot of individual producing well flow responses of the upscaled model with respect to the truth model for three reservoir test cases (Reservoir A, B, C are represented by column 1, 2, 3 respectively). (**a**) Water breakthrough time (Years). (**b**) Oil production rate (Sm^3^/day) at water breakthrough time (black circles) and at 20 years of simulation (grey circles). (**c**) Water-cut (fraction) at 10 years (black triangles) and at 20 years of simulation (grey triangles).

**Figure 5:**
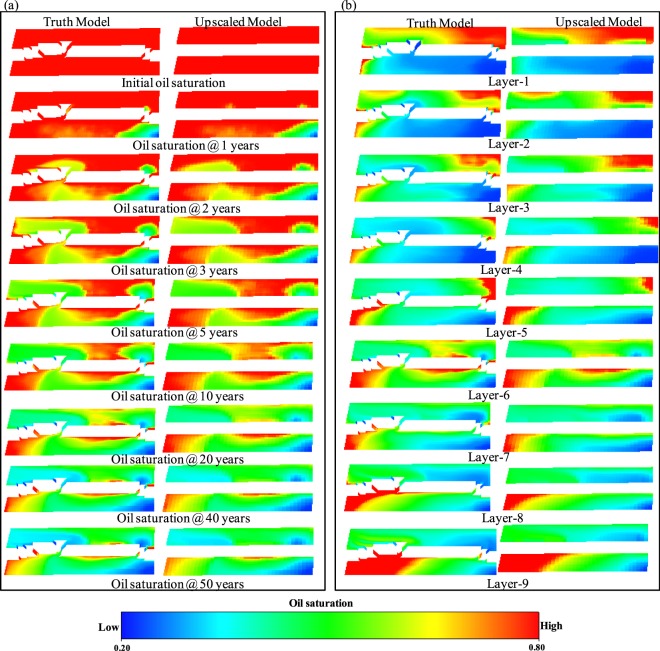
Saturation maps of reservoir A. (**a**) Layer 6 for different time steps. (**b**) All layers at 20 years of simulation.

**Figure 6:**
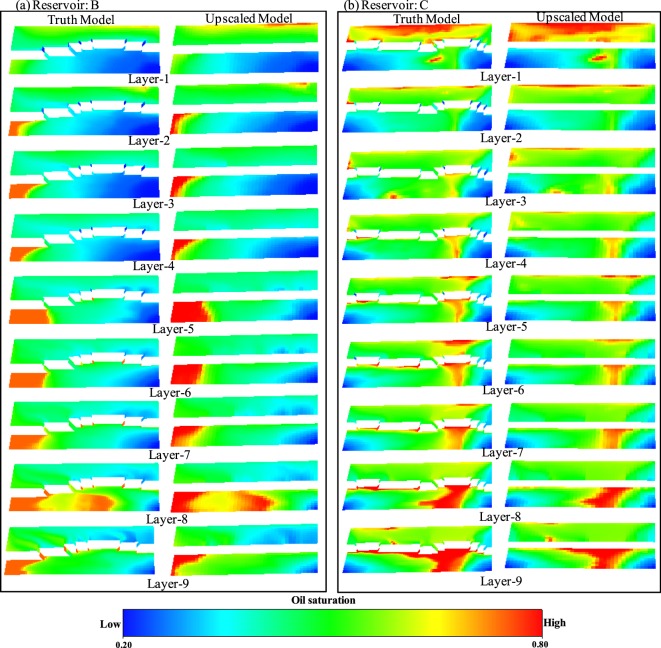
Saturation maps at 20 years of simulation for all the nine layers of the truth model and the upscaled model. (**a**) Reservoir B. (**b**) Reservoir C.

The accuracy of the newly devised FBGU method is tested by comparing the flow results of the truth model with those of the upscaled model for three synthetic test reservoirs (Table 1) in terms of reservoir performance (Fig. 3), individual layer or well performances (Fig. 4), and cell oil saturation through time (Figs 5 and 6). These are discussed in turn. Throughout this Section, the version of Reservoir A with a k_v_/k_h_ ratio of 0.0001 is used. The version with the higher k_v_/k_h_ ratio (for reservoir A) is used to discuss the influence of vertical permeability at the end of this Section. Note that flow simulation modelling on a high-resolution truth model is beyond the scope for a full-field real reservoir model; however it is indispensable in this study to validate the newly developed FBGU method.

### Reservoir performance

The equivalent sets of graphs for the truth model and the upscaled model data are reported for the three test reservoirs (Fig. 3). The average reservoir pressure, oil production rate, cumulative oil production, and water-cut are plotted as a function of simulation time, and all show a close similarity between the overall flow responses of the upscaled models (grey coloured solid line) and the truth models (black coloured circles) in each test reservoir. There is a slight tendency for the total oil production to be lower and the average reservoir pressure to be higher, in the upscaled model as opposed to the truth model for reservoir C, which is discussed in the subsequent Section. Overall, however, these results imply that the newly devised FBGU method is an accurate way of representing sub-seismic fault zones into low-resolution upscaled models.

### Well performance

To test the FBGU method more precisely, individual layer or well responses (i.e. time to water breakthrough, and oil production rate and water-cut for each well at different time steps) of the upscaled model are compared with those of the truth model for three test reservoirs (Fig. 4). Oil production rate is plotted at the time to water breakthrough (WBT) and at 20 years of simulation, and water-cut is plotted at 10 years and at 20 years of simulation. The individual well responses of the models especially the time to water breakthrough (Fig. 4) are a bit different in each reservoir test case. The inconsistencies might have been introduced during the FBGU process, during two-phase numerical flow simulation modelling to test the method, or due to the petrophysical property upscaling processes from geological models. However, the FBGU method gives a good result in terms of individual well for the three reservoir test scenario.

### Saturation maps

A visual inspection of the saturation maps (Figs 5 and 6) is another means of comparing the upscaled and truth models. Figure 5a shows that the saturation maps of layer 6 of the truth model A and the upscaled model of reservoir A are very similar to each other at different time steps, while Fig. 5b shows this for all layers at a particular time (e.g. @20 years). Figure 6 shows the saturation maps of each layer of the models B & C. These figures reveal that the saturation profile of the upscaled models of three reservoir test cases are very similar to the corresponding truth models, implying that the newly developed FBGU method is an accurate means of representing sub-seismic fault zone components into upscaled flow simulation models.

### Effects of vertical permeability

**Figure 7:**
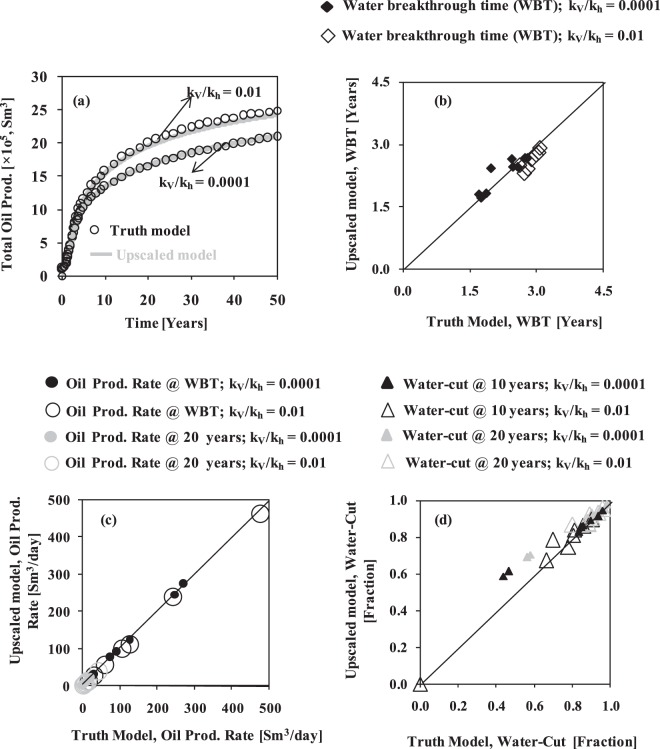
The influence of vertical permeability in the FBGU method. (**a**) The total oil production for two permeability anisotropy values (top: k_v_/k_h_ = 0.01, and bottom: k_v_/k_h_ = 0.0001). (**b**) Water breakthrough time (Years). (**c**) Oil production rate (Sm^3^/day) at water breakthrough time (black) and at 20 years of simulation (grey). (**d**) Water-cut (Fraction) at 10 years (black) and 20 years of simulation (grey). The filled markers are for k_v_/k_h_ = 0.0001, and the hollow markers are for k_v_/k_h_ = 0.01.

Reservoir A has been selected to check the influence of permeability anisotropy (i.e. k_v_/k_h_ ratio) on the accuracy of the FBGU method. The FBGU on the isolated fault zone component (discussed in Method Section) and the two-phase flow responses of model A is performed for two permeability anisotropy values of 0.0001 (lower) and 0.01 (higher). The two-phase numerical flow responses for the lower k_v_/k_h_ value were discussed in the previous sections (Figs 3–6). The effect of permeability anisotropy is tested by comparing the flow results of the truth model and the upscaled model in terms of field cases (Fig. 7a) and the individual well responses (Fig. 7b–d). Figure 7a shows that the flow response of the upscaled model in terms of hydrocarbon production is very similar to the truth model for the lower k_v_/k_h_ ratio case, but less for the higher k_v_/k_h_ ratio case, which shows a similar slight underestimation of total oil production (like Reservoir C, Fig. 3). The individual well performances in terms of the time to water breakthrough, oil production rate and water-cut also show a good agreement between the upscaled model and the truth model even for the higher k_v_/k_h_ ratio case (Fig. 7b–d). The water breakthrough time of each corresponding producing well happens slightly earlier in the upscaled model than in the truth model for higher k_v_/k_h_ value. This combination of slightly lower oil production (Fig. 7a) but slightly earlier water breakthrough (Fig. 7b) suggests that the flow paths from injector to producer are slightly more direct in the upscaled model as opposed to the truth model. This could be due to an inaccuracy in the upscaling, but could equally be because the two models have different resolutions, as indicated by a comparison of Fig. 2c–d. Overall, the results demonstrate a remarkable similarity between the truth models and upscaled versions, confirming the conclusion of Islam and Manzocchi^26^ that the new FBGU approach developed in this research is significantly better than the earlier TBGU approach^18^.

## Conclusions

This section summarises the reasons why further research to represent the complex 3D fault zone structures into an upsaled model by devising a new FBGU method is indispensable. From a modelling perspective, local grid refinement (LGR) technique has never been demonstrated within a full-field flow simulation model. Therefore, the principal aim of the present study has been to improve a particular method to represent the sub-seismic fault zones into the field-scale flow simulation models, referred as geometrical upscaling (GU). GU can easily be applied to conventional upscaled flow simulation models by assigning new connection transmissibilities between cells as the across-fault and up-fault flow paths without altering the geometrical configuration of the conventional upscaled flow models. This is the reason why GU is considered as a particular flexible means of representing the sub-seismic fault zone components into flow simulation models.

Another important aim of this study was to overcome the limitations of the existing TBGU method. A new FBGU algorithm has been applied to upscale models containing complex sub-seismic fault zone structure (Fig. 2c) to the resolution of a conventional upscaled flow simulation model (Fig. 2d). It would not be possible to upscale these relays using the existing TBGU and LGR method. This newly developed FBGU method has been tested for three synthetic reservoir models, which contain realistic small-scale fault zone complexity.

The application of this new FBGU method provides good results in terms of individual well responses and results of field cases for the upscaled models in each of three test reservoirs for different permeability anisotropy values. Therefore, the newly devised FBGU algorithm is a flexible and an accurate means of representing complex small-scale sub-seismic fault zone geometry into low-resolution conventional upscaled production models regardless of the structures, size and location of the fault zone components, and regardless of the petrophysical property heterogeneity and permeability anisotropy.

## Methods

Working principle of the novel flow-based geometrical upscaling (FBGU) approach. The existing TBGU method explained why the use of a template makes the method inflexible and difficult to extend (please see the Supplementary Notes 1 and 2 for TBGU method). A new and emerging approach, referred as FBGU is therefore formulated in this study to simplify the existing TBGU approach and to make it more flexible. In this FBGU method, all cell-centre to cell-centre connection transmissibilities between simulation model cells through the fault zone are calculated by conducting single-phase, incompressible steady state numerical flow simulation modelling within a high-resolution truth model which contains the 3D fault zone structure explicitly. In this flow simulation model, the injector and producer wells are controlled by well bottom hole pressure ensuring a constant pressure drop (i.e. ΔP) between the cells and a steady state volumetric flow rate (Q). The transmissibility of the flow path between the cells containing injector and producer wells is deduced from these values and the viscosity of the flowing fluid (µ) using the simplified Darcy’s law, which can be written as:

1 $$Q=\frac{kA}{L}\times \frac{{\rm{\Delta }}P}{\mu }$$

where Q is the volumetric flow rate, A is the contact area, k is the equivalent permeability of the two grid cells, ΔP is the pressure difference between the injector and producer wells, μ is the viscosity and L is the distance between the two cell centres over which pressure drop is taking place.

The flow simulator simplifies eq. (1) by combining all the terms that do not change over the course of a simulation run, to give:

2 $$Q={T}_{12}\times \frac{{\rm{\Delta }}P}{\mu }$$

where T_12_ is the transmissibility between the centres of the two grid cells.

Therefore, the simplified general form of the transmissibility between the two grid cells can be re-written by combining the eqs (1) and (2) as:

3 $${T}_{ij}=\frac{{Q}_{j}.\mu }{{P}_{i}-{P}_{j}}$$

where subscript i represents the cell containing the injection well, j represents the cell containing the producing well, T is the transmissibility between the cells containing injection and producing wells, Q is the volumetric flow rate of the flow path recorded in the producing well, µ is the viscosity of the flowing fluid (water in this case), and P_i_ & P_j_ are the bottom hole pressures of the injector and producer wells respectively.

**Figure 8:**
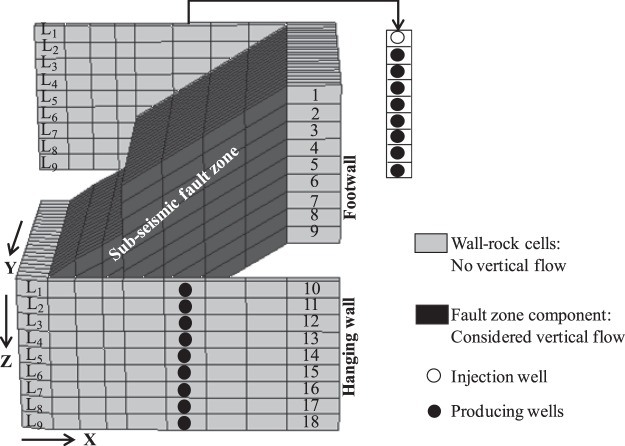
Example figure of a breached fault zone component with the adjacent wall-rock cells in 3D to explain the newly devised FBGU method. One of 18 well configurations applied in the upscaling is shown. Vertical no flow boundary conditions are applied to the wall-rock cells on either sides of the fault indicated by grey colour to ensure the fluid flow between the wall- rock cells through the fault zones only.

The next section considers the FBGU for a 9 layers fault zone component model (Fig. 8) and the across-fault and up-fault transmissibilites associated with 18 cells from both sides of the fault can be estimated by the new FBGU method. In the FBGU method, these transmissibilities are determined by placing an injector well in any of the wall rock cells, and 17 producer wells in each of the other 17 cells (Fig. 8), and deducing the transmissibility from the flow simulation results. This process is repeated 17 more times by changing the location of the injector well to the remaining 17 cells in turn to define the complete set of transmissibilities associated with the model. The process is described in detail in the next Section.

Upscaling of a single fault zone component. Figure 8 is a complex and realistic fault zone component in truth model in 3D. The grey coloured cells are the adjacent wall-rock cells on either side of the fault zone, and the black coloured cells are the sub-seismic fault zone component. To ensure that only flow paths between the wall-rock cells through the fault zone are calculated and output by the upscaling algorithm, no flow boundary conditions in the vertical direction are applied to the wall-rock cells on the footwall and hanging wall sides of the fault, even if vertical flow is possible between these cells in the truth model. In contrast to the existing TBGU approach, however, the model vertical permeability is honored in the fault zone cells.

A fault zone model with 9 active layers has a total of 18 layers from both sides of the fault (Fig. 8). The wall-rock cell stacks closest to the centre of the ramp are chosen, and all the possible across-fault and up-fault connection transmissibilities between the 18 cells within these stacks on either side of the fault, through the fault zone, are calculated based on the working principle of FBGU method described in the previous section. This involves conducting incompressible steady state high-resolution numerical flow simulation modelling on the fault zone component (Fig. 8) using 18 different flow simulation models.

Consider flow from the top layer in the footwall (i.e. i = 1) to all other remaining 17 grid cells (i.e. j = 2 to 18) on either side of the fault as illustrated in Fig. 8. This requires one injector well (black coloured hollow circle) and 17 producer wells (black coloured filled circle) for calculating the 17 possible connection transmissibilities, which are associated with the cell containing the injector well (Fig. 8). These 17 possible connection transmissibilities through the fault zone are calculated using the simplified general form of Darcy’s law (eq. (3)) based on multipoint flux approximation (MPFA)^40–43^ and are given by:

4 $$\begin{array}{cccc}{T}_{1,2}=\frac{{Q}_{2}.\mu }{{P}_{1}-{P}_{2}}, & {T}_{1,3}=\frac{{Q}_{3}.\mu }{{P}_{1}-{P}_{3}}, & {T}_{1,4}=\frac{{Q}_{4}.\mu }{{P}_{1}-{P}_{4}}, & {T}_{1,5}=\frac{{Q}_{5}.\mu }{{P}_{1}-{P}_{5}}\\ {T}_{1,6}=\frac{{Q}_{6}.\mu }{{P}_{1}-{P}_{6}}, & {T}_{1,7}=\frac{{Q}_{7}.\mu }{{P}_{1}-{P}_{7}}, & {T}_{1,8}=\frac{{Q}_{8}.\mu }{{P}_{1}-{P}_{8}}, & {T}_{1,9}=\frac{{Q}_{9}.\mu }{{P}_{1}-{P}_{9}}\\ {T}_{1,10}=\frac{{Q}_{10}.\mu }{{P}_{1}-{P}_{10}}, & {T}_{1,11}=\frac{{Q}_{11}.\mu }{{P}_{1}-{P}_{11}}, & {T}_{1,12}=\frac{{Q}_{12}.\mu }{{P}_{1}-{P}_{12}}, & {T}_{1,13}=\frac{{Q}_{13}.\mu }{{P}_{1}-{P}_{13}}\\ {T}_{1,14}=\frac{{Q}_{14}.\mu }{{P}_{1}-{P}_{14}}, & {T}_{1,15}=\frac{{Q}_{15}.\mu }{{P}_{1}-{P}_{15}}, & {T}_{1,16}=\frac{{Q}_{16}.\mu }{{P}_{1}-{P}_{16}}, & {T}_{1,17}=\frac{{Q}_{17}.\mu }{{P}_{1}-{P}_{17}}\\ {T}_{1,18}=\frac{{Q}_{18}.\mu }{{P}_{1}-{P}_{18}} &  &  & \end{array}$$

The flow simulation model assumes incompressible and steady state flow, the flowing fluid, therefore, conforms to the laws of conservation in numerical flow simulations as:

5 $${Q}_{i=1}=\sum _{j=2}^{18}{Q}_{j}$$

where Q_i_ is the flux of injection well, Q_j_ is the flux of different individual producing wells through the fault zone.

These 17 transmissibilities (eq. (4)) are calculated from a single run of simulation model and are all associated with a particular wall-rock cell (i.e. cell 1). To get the remaining possible connection transmissibilities from the other 17 remaining wall-rock cells on either side of the fault in Fig. 8 (i.e. cells 2 to 18), 17 more simulations are required. Therefore, a total 18 numerical flow simulations are required (which is equal to the total number of wall-rock cells from both sides of the fault) to get all the possible connection transmissibilities through the fault zone. Hence, the total number of possible transmissibilities between 18 wall-rock cells is 18 × (18-1) in this case which can be represented algebraically as ^18^P_2_. A 18 × 18 matrix is formed to get all of the possible flow paths and is represented by eq. (6).6In eq. (6), the diagonal components of the matrix are the flow paths that start and end in the same cell, and therefore are not relevant. Hence, all the possible across-fault and up-fault connection transmissibilities are the off-diagonal components in the matrix (eq. (6)). The seventeen (17) off-diagonal components in the first row/column in eq. (6) are calculated from one simulation run associated with the injector well in cell 1 (Fig. 8), and the off-diagonal components of the remaining 17 rows/columns are computed in a similar way using another 17 numerical flow simulations with the injector in a different cell in each case.

In eq. (6), each off-diagonal matrix component exists twice in the matrix, one in the upper triangular matrix (solid black coloured triangle) and the other one is in the lower triangular matrix (dotted black coloured triangle). These off-diagonal matrix components are symmetrical and have equal numerical value to each other (i.e. T_1,2_ = T_2,1_ …. T_17,18_ = T_18,17_). Since each off-diagonal component exists twice in the matrix and has an equal numerical value, the total number of possible connection transmissibilities for the upscaled model will be half of the total number of off-diagonal connections that exist in the matrix. Therefore, the off-diagonal matrix components either from the upper triangular matrix or from the lower triangular matrix are assigned as neighbour and non-neighbour connection transmissibilities to the upscaled model. The upscaling of a fault zone component model (Fig. 8) described above a 9-layer model; if such a model has N layers, then total across-fault and up-fault transmissibilities associated with 2N cells from either side of the fault would be 2N(2N-1), which could be deduced from 2N number of flow simulation models. Therefore, FBGU method described above for a 9-layer fault zone model is the generalized form of upscaling a fault zone model containing any number of layers in question.

It is important to appreciate the role that the vertical permeability of the fault zone cells has on the upscaled results. For example, in the case of Fig. 8, there is no direct flow path through a single layer of the relay ramp between hanging wall layer 9 (i.e. cell 18) and footwall layer 1 (i.e. cell 1), and the existing TBGU approach of Manzocchi *et al*.^18^ would not output a connection between these cells. In the FBGU approach, however, vertical flow is possible within the ramp, and so a pressure response is observed in producer cell 18 from injector in cell 1, and from this a transmissibility is calculated.

As mentioned previously, the wall-rock cells are assigned zero vertical permeability in the upscaling irrespective of their input value, as only flows within the fault zone are required during the upscaling. Vertical flow through the cell stack outside the fault zone will be simulated during the full-field simulation to test the FBGU method (Supplementary Figs S3 and S4). Note that the FBGU method described above is for a one fault zone model that is isolated from the high-resolution truth model (Fig. 2c) in which petrophysical properties (e.g. permeability, porosity, net-to-gross) for each of wall-rock and fault zone cells are very heterogeneous. However, in the model described above (Fig. 8), the wall-rock cells are coloured as grey and fault zone cells are coloured as black only to make differentiation between them and to show that there is no vertical flow between wall-rock cells during the upscaling process but it is honoured within fault zone cells. The isolation of fault zone components from the high-resolution truth model (Fig. 2c) is described in the next section.

### Isolation of fault zone components

**Figure 9:**
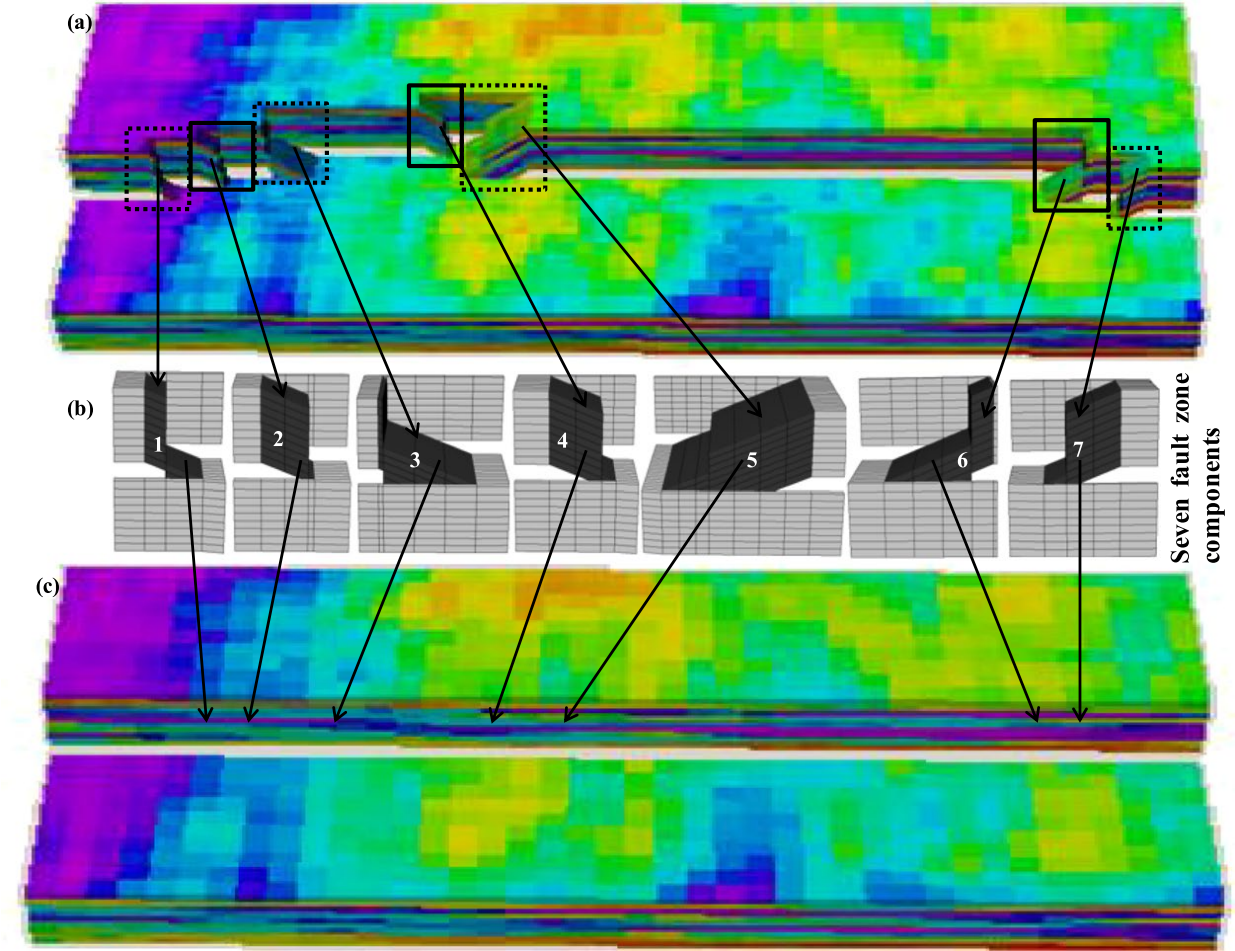
Geometry of reservoir model A. (**a**) The truth model contains seven explicit sub-seismic fault zone components. (**b**) Individual fault zone components are coloured black and their adjacent wall-rock cells are coloured grey to make differentiation between them, however, all cells of wall-rock and fault zones contain heterogeneous petrophysical properties of truth model. All other cells are rendered inactive during the upscaling of each component. (**c**) The components are upscaled and represented by connection transmissibilities at the closest equivalent location into the low-resolution upscaled model.

The most important and foremost fundamental task for conducting the FBGU of sub-seismic fault zone components is to isolate all the fault zone components available in the high-resolution truth model into different regions with their corresponding adjacent along-fault and across-fault wall-rock cells (Fig. 9). The high-resolution truth model shown in Fig. 2c is considered as an example reservoir model to describe the isolation process. The isolation of sub-seismic fault zone components of a reservoir model is done manually (using the standard Eclipse^32^ ACTNUM keyword) and each component is subsequently dealt with individually. Eclipse^32^ ACTNUM keyword assigns two numbers (e.g. either 0 or 1) to all cells of a reservoir model to isolate a fault zone component. The number 0 is assigned for all cells that would like to be inactive and 1 is assigned for all active cells. For example, if a modeller is interested to isolate the first fault zone component (Fig. 9b), ACTNUM must assign a value of 1 to all cells within the first bounded black box and 0 value to the remaining cells of the model. In the test model (Fig. 9a), the seven available fault zone components must be isolated individually into seven different components as shown in Fig. 9b. The method described above is applied to each component in turn, and the output transmissibilities are assigned to cells in approximately at the equivalent location in the low-resolution upscaled model (Fig. 9c). The inclusion of neighbour and non-neighbour connection transmissibilities into upscaled model is presented in the Supplementary section (please see Supplementary Note 4).

An important consideration is the decision of how much of the model surrounding the fault zone component should be included in the isolated fault zone model. For the implementation of the method discussed by Islam and Manzocchi^26^, the width of the isolated region was equivalent to the width of a cell in the upscaled model, which seems intuitively an appropriate value. In the implementation discussed in this study, the decision was made to include one cell in each horizontal direction in the isolated fault zone component model (Figs 8 and 9b). Cells in FaultMaker tend to be of variable size parallel to the fault (i.e. in the X-direction) and very narrow perpendicular to it (Figs 8 and 9b), and therefore the resultant boundary size is variable, and relatively arbitrary. Despite this, this study will show that the results of these models are accurate. Assessment of the optimum volume to include in the isolated region is beyond the scope of this study, and may be the subject of future work.

### Summary of FBGU approach

The methodology to upscale the sub-seismic fault zone components within the truth model using the new FBGU method (i.e. the stage 6 of modelling workflow Section, described thoroughly in this Section) is sub-divided, and summarised below.

Stage 6.1 Isolate the fault zone components with their adjacent wall-rock cells into different individual regions.

Stage 6.2 Along the fault zone length, identify the approximate mid-point of the non-uniform wall-rock cells adjacent to fault zone component for placing the injector and producer wells (Fig. 8).

Stage 6.3 Apply the FBGU algorithm described in this Section to upscale the explicit sub-seismic fault zone components available in the truth model (Fig. 2c) as a function of neighbour and non-neighbour connection transmissibilities, which are represented implicitly into the low-resolution upscaled model (Fig. 2d).

Stage 6.3 is repeated 2 N times (for a fault zone component model containing N layers), with the injector well location in a different layer on both sides of the fault in each flow simulation model.

Stages 6.2 and 6.3 combined are repeated for each fault zone component present in the truth model. Hence in the example truth model (Fig. 9a, which has 7 fault zone components and 9 layers), a total of 126 flow simulation runs are required to upscale the 7 isolated fault zone components.

## Supplementary information

Supplementary Information

## Data Availability

The datasets that support the plots within this paper and other findings of this study are available from the corresponding author upon reasonable request.
