# Supplementary material for: A novel flow-based geometrical upscaling method to represent three-dimensional complex sub-seismic fault zone structures into a dynamic reservoir model
Source: Sci Rep. 2019 Mar 28;9:5294. doi: 10.1038/s41598-019-41723-y (PMC6438981; doi:10.1038/s41598-019-41723-y)
Supplement: Supplementary file 1 — Supplementary Information [file 41598_2019_41723_MOESM1_ESM.pdf]

A novel flow-based geometrical upscaling method to represent three-dimensional complex sub-seismic fault zone structures into a dynamic reservoir model

Md Saiful Islam[1, 2*], Tom Manzocchi[1]

Supplementary Note 1

Background of GU to modelling fault zone structures

Discrete sub-seismic fault zone components (e.g. breached and unbreached relay zones) may be present at different locations on a fault, but are difficult to incorporate into flow simulation models explicitly due to their size (small compared to upscaled model's grid block size), resolution (below the seismic data), and very complex geometry. GU solves this issue by representing complex sub-seismic fault zone structure implicitly into the flow simulation model as a function of connection transmissibilities that cannot be visualised at the resolution of the flow simulation model. Therefore, GU is referred as a connectivity-based algorithm that aims to upscale structurally complex 3D sub-seismic fault zone components to the resolution of a upscaled flow simulation model[18,28,44]. The objective of the GU method is to estimate transmissibilities of different up-fault and across-fault flow paths present through a fault zone, and to include them as neighbour and non-neighbour connection tramsmissibilities in the low-resolution full-field flow simulation model in which the fault is represented as a 2D planar surface. In the GU approach, the connection transmissibilities are calculated from the centre of one cell to the centre of another cell associated with complex 3D fault zone structure (Supplementary Fig. S1a-b) and representing them into the upscaled flow model (Supplementary Fig. S1c). The GU method first devised by Manzocchi et al.[18] is a TBGU approach and implemented in TransGen software[30], has rarely been applied in real reservoir studies documented in the public domain (an exception being[45]). Geometrically, the method has constraints in terms of length and complexity of the fault zone structures that can be considered,

[1]Fault Analysis Group, Science Centre West, School of Earth Sciences, University College Dublin, Ireland. [2]Present Address: Department of Mechanical and Mechatronics Engineering, College of Engineering, Dhofar University, Salalah, Oman. *E-mail: mislam@du.edu.om

and the petrophysical properties within the zones. Specially, this TBGU method is applicable only for fault zone structure contained within two-cell stacks (Supplementary Fig. S1), where the fault zone is smaller than a single grid cell and can't deal with anisotropic petrophysical properties and two-phase fault rock properties. Therefore, the principal objective of this study is to devise a novel FBGU approach to expand the flexibility of the existing TBGU method in terms of geometries, scales and properties that geometrical upscaling is capable of dealing with. Moreover, the upscaling algorithm devised by Manzocchi et al.[18] contains numerous assumptions and simplifications, and has never been tested rigorously. In the existing TBGU approach, the connection transmissibilities are estimated geometrically[18,28,30], but in the newly developed FBGU method described (see method section) in this study is more accurate and more flexible[26], it is done by conducting steady state numerical flow simulation. The following supplementary note 2 represents a brief discussion and limitations of existing TBGU approach to justify the development of the new FBGU approach for the modelling of realistic and structurally complex fault zones.

Supplementary Note 2

Overview of the existing TBGU approach and its limitations

In the existing TBGU approach[18], the fault zones are upscaled as a function of connection transmissibility which is calculated from the complex 3D fault zone geometry (Supplementary Fig. S1a), and reformatted as neighbour and non-neighbour connections for the upscaled flow simulation models (Supplementary Fig. S1c). To describe the existing TBGU method, consider an extremely simple model consisting of two-cell stack with 9 layers of which only 5 are active (Supplementary Fig. S1a). The model contains a double breached relay ramp, which is smaller than the grid cells. A 2D Allan diagram is illustrated to see the connectivity of different layers of two grid cells through the fault zone (Supplementary Fig. S1b). In this example model (Supplementary Fig. S1a-b), consider flow from the fifth cell in the footwall (FW5) to all other cells in the foot wall and hanging wall of the breached relay model through the fault zone. The existing TBGU algorithm can calculate transmissibilities between the reference footwall cell (FW5) and 2 cells in the footwall (FW7, FW9) and 3 cells in the hanging wall (HW1, HW3, HW5). Hence, there are five possible connection transmissibilities are available of which one is

across-fault neighbour (e.g. the connection: FW5-HW5), two are up-fault non-neighbour (e.g. connections: FW5-FW7, FW5-FW9), and two are across-fault non-neighbour (e.g. connections: FW5-HW1, FW5-HW3) connections. However, two more transmissibilities are ignored in the existing TBGU method, and is discussed latter in this section. These five transmissibilities are then included as neighbour and non-neighbour connections in the low-resolution upscaled model (Supplementary Fig. S1c), to give the same flow paths. The cells immediately above and below the cell of interest are not included as flow paths in the workflow of Manzocchi et al.[18] and therefore these cells are considered as inactive in this model, this is discussed further later in this section. The scale of the task can be put in context by appreciating that these five connection transmissibilities are associated with one cell, and the other 9 cells from either side of relay of these two cell stacks must be considered for the estimation of across-fault and up-fault transmissibilities through the fault zone in a similar way to get the complete set of flow paths. In this approach, the connection transmissibilities are calculated as a function of the geometry, contact area, and properties of the cells[18,30,32].

There are few limitations in the existing TBGU algorithm to handle fault zone structures in various contexts. The first class of limitation is associated with the template that underlies the method. In this TBGU method, the template allows consideration of only a limited subset of possible sub-resolution fault zone structure (Supplementary Fig. S2b). Whilst it is possible to extend the range of templates, it is impractical to design a sufficiently wide range of templates to handle every possible eventuality (Supplementary Fig. S2c-d). The second class of limitation is that certain flow paths are ignored. For example, flow paths between the reference footwall cell (FW5) and 2 cells in the footwall above the reference cell (e.g. connections: FW5-FW1, FW5-FW3) are ignored, however, these are possible through layers 1 and 3 of the relay ramp. Another important limitation is associated with the vertical permeability within the fault zone, which is ignored. This means paths that pass vertically between layers in the ramp are ignored. This decision was, presumably, made to simplify the method, but has not been validated or justified. All these classes of limitations are addressed using the newly developed FBGU method that is applied to a more complex fault zone. A final limitation of the method is that only single-phase fault rock properties can be included. GU including two-phase fault rock properties was beyond the scope of what was considered by Manzocchi et al.[18], but the new FBGU workflow for doing this is presented by Islam and Manzocchi[31].

Overall, however, the existing TBGU method shows that the whole technique relies on defining a template for each type of fault zone component; it cannot be flexible for larger and more complex fault zones. For example, the template devised for two cell stacks is inappropriate for a relay ranging over four or more cell stacks, which would need a completely new template. Therefore, an entirely different approach that does not rely on templates is desirable. A new FBGU method is devised to make the GU more flexible and is presented in this study.

Supplementary Note 3

Summary of the models for testing the method

Three different test models called reservoir **A**, **B**, and **C** are used in this study to test the FBGU algorithm (Supplementary Fig. S3). The dimension, resolution and geometry of the models are shown in Table 1. Reservoir model **A,** with seven breached relay ramps (Supplementary Fig. S3a), and model **C** with eight breached relay ramps (Supplementary Fig. S3c) both have total throws of 110m which is larger than the total 90m model thickness, therefore, there would be no communications between the injector and producer wells without these fault zone components. Model **B**, by contrast, contains six breached relay ramps (Supplementary Fig. S3b) and has a total throw of 80m, which is smaller than the total 90m model thickness and therefore across-fault fluid flow would be possible even in the absence of the relay zones. Supplementary Fig. S4 shows the upscaled models for which the FBGU has been performed (described in Method Section) to include the effects of the relay zones.

The horizontal permeability is modelled isotropically (i.e. k_x=k_y) as shown in Supplementary Figs S3 and S4. Vertical permeability is modelled using a k_v/k_h ratio. Reservoir **A** is tested for two different k_v/k_h ratio values of 0.0001 and 0.01, and Reservoirs **B** & **C** are modelled using a k_v/k_h ratio value of 0.001.

The models are initially oil saturated, and a water-flood is simulated using the well locations shown in Supplementary Figs S3 and S4. The vertical water injection and vertical oil producing wells are perforated throughout the formation for both Reservoir **A** & **B**. The injection and the producing wells are located at opposite corners of the models. Reservoir **C** has two vertical injection wells and two vertical producing wells located at the four corners of the model.

The two injection wells and the two producing wells are all perforated throughout the formations and therefore the production is co-mingled from all layers.

Water and oil relative permeability curves used in this study are calculated using the equations of Christie and Blunt[46], and are shown in Supplementary Fig. S5.

The water PVT properties of the models are: B_w= 1.05, c_w = 3.10^{-6} bar^{-1}, μ_w = 0.45 cp. The dead oil PVT properties are reported in Supplementary Table S3.

The top of three test models is 1000m deep. Therefore, the reference depth is 1100m for reservoir A and C, 1085m for reservoir B and the initial pressure at reference depth is considered of 1000 bar. Surface oil, and water densities are 876 kg/m^3, and 1000 kg/m^3 respectively and the rock compressibility is 10^{-6} bar^{-1}. The well bore diameter is considered as 0.25m in this study.

The injection wells are constrained by a water injection rate of 1000 rm^3/day, while the producing wells are controlled by fixed well bottom hole pressure (BHP) of 300 bar for each reservoir test cases. All the simulations are run for 50 years of production.

Supplementary Note 4

Inclusion of connection transmissibilities into a upscaled model

In this study, the Eclipse flow simulator[32] is used in the upscaling process. Therefore, the Eclipse simulator requires different keywords for representing neighbour or non-neighbour connection transmissibility, and the procedure for including them into flow simulator is discussed here. In a corner point grid (CPG) model, a connection between two cells is termed a neighbour connection if a single digit in all co-ordinates separates the two cells. Hence, the cell [I, J, K] is neighbour to cells [I-1, J, K], [I+1, J, K], [I, J-1, K], [I, J+1, K] in the two horizontal directions, and to cells [I, J, K-1], [I, J, K+1] in the vertical direction. Whereas tortuous flow paths through the fault zone might form across-fault and up-fault non-neighbour connection transmissibilities (e.g. FW5-FW3 is an up-fault and FW5-HW1 is an across-fault non neighbour transmissibilities, Supplementary Fig. S1a-b).

For faulted neighbour connections, the implicit transmissibility values calculated through the fault zones are added to the explicit transmissibility values approximated by simulator without the fault zones, and include as TRANX, TRANY and/or TRANZ keywords in the EDIT

section of the Eclipse input file to define the complete neighbour connections. In the Supplementary Fig. S1a, the neighbour connection between the reference cell in the footwall (FW5) and the fifth cell in the hanging wall (HW5) is the implicit transmissibility as these two cells are not explicitly connected for this particular model. This transmissibility is included in Eclipse using the TRANX keyword. An example format of neighbour connections (e.g. TRANX) is given below.

BOX

--I_1-I_2 J_1-J_2 K_1-K_2

1 1 1 1 5 5/

TRANX

0.05/

For faulted implicit non-neighbour connections, the NNC keyword in the GRID section is used to define non-neighbour connection transmissibility. An example format of the non-neighbour connection is given below considering Supplementary Fig. S1a (the connection between the reference cell in the footwall, FW5 and the first cell in the hanging wall, HW1).

NNC

--$Cell_1$	$Cell_2$	TRAN
1 1 5	2 1 1	0.035/

/

Beside these if explicit non-neighbour connections that could be formed without the fault zone are available in the model, the EDITNNC keyword is used in the EDIT section to define as a transmissibility multiplier. An example format of this keyword is:

EDITNNC

--$Cell_1$	$Cell_2$	TRANM
1 1 10	2 1 7	0.0035/
1 1 11	2 1 7	0.0055/
1 1 12	2 1 7	0.0030/

/

Between them, these keywords are able to represent the range of connections produced by sub-seismic fault zone component.

Supplementary Figures

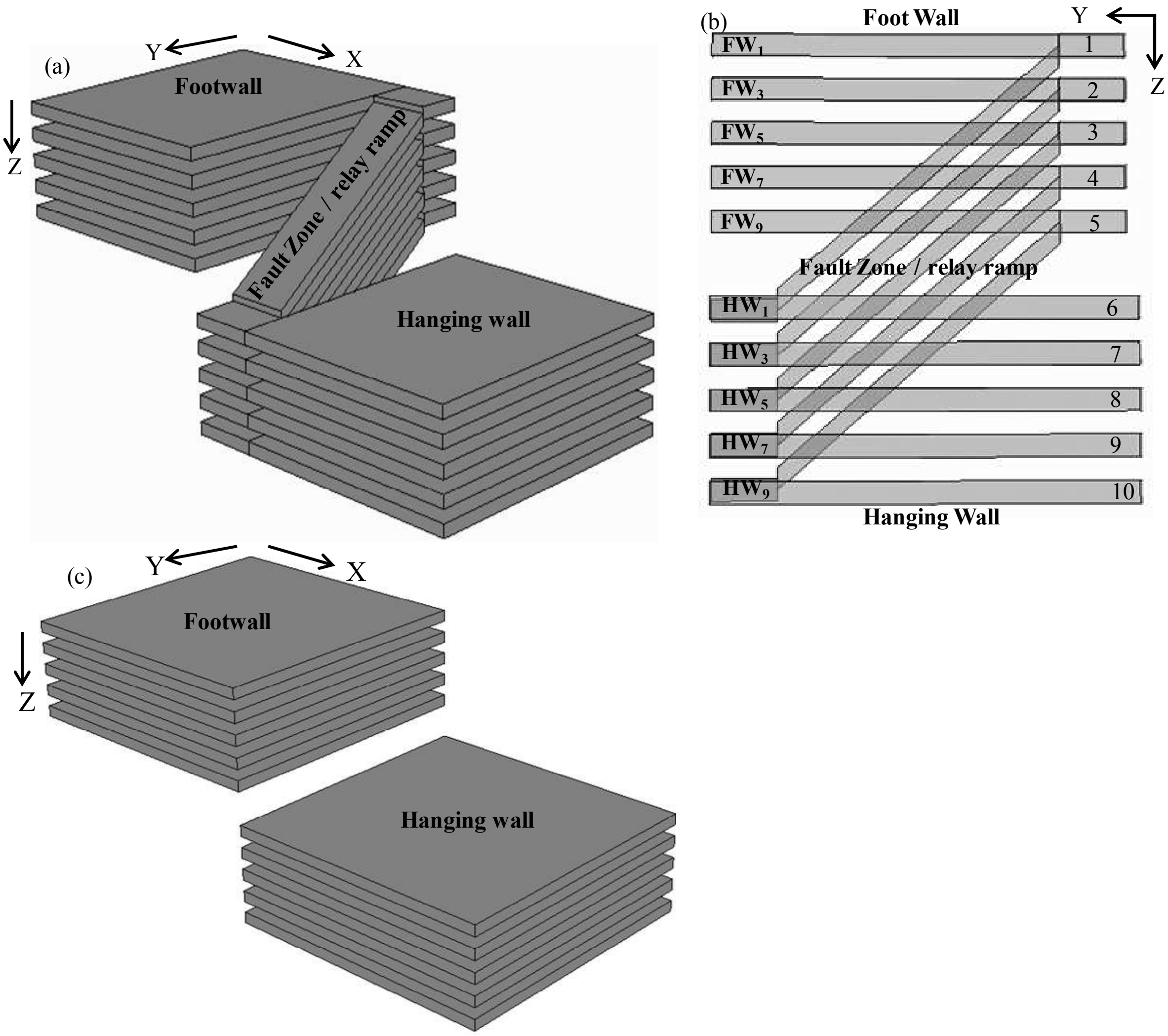

Supplementary Figure S1. Example of a model built in two-cell stacks containing 3D explicit fault zone, used to describe the existing TBGU method. (**a**) 3D geometry of the model contains a breached relay ramp as 3D fault zone structure. (**b**) 2D Allan diagram represents the connectivity between the two cells through the ramp. (**c**) low-resolution upscaled model version of (a), which contains the same fault zone structure from the truth model implicitly as a function of non-neighbour and neighbour connection transmissibilities.

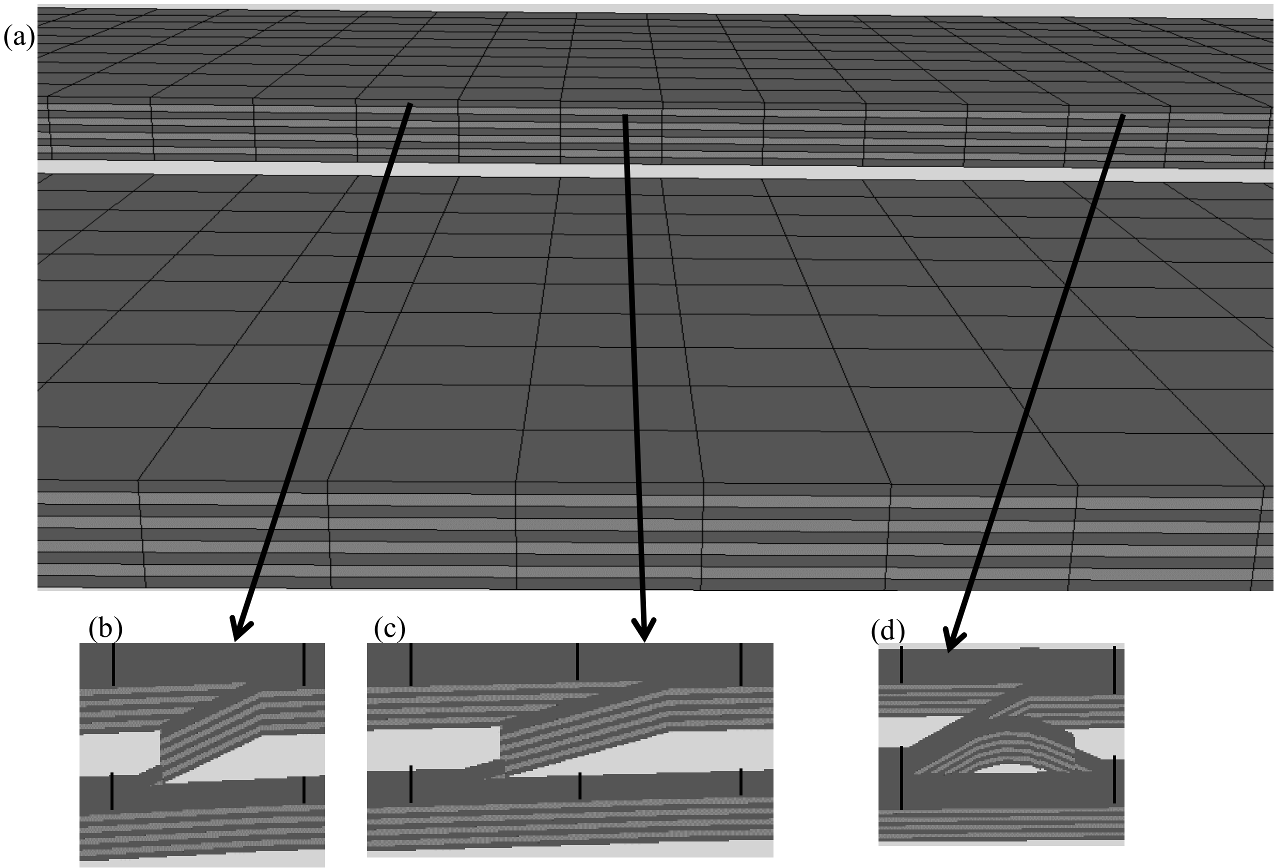

Supplementary Figure S2. (**a**) Portion of a faulted full-field simulation model. (**b**) Geometrically simple relay zone that can be upscaled using the existing TBGU method. (**c**) Relay zone that cannot be upscaled using the existing TBGU method as it extends over more than a single grid cell. (**d**) Fault zone that cannot be upscaled using the existing TBGU method as it is geometrically too complex for the simple template. N.B: black lines indicate the edges of the grid cells.

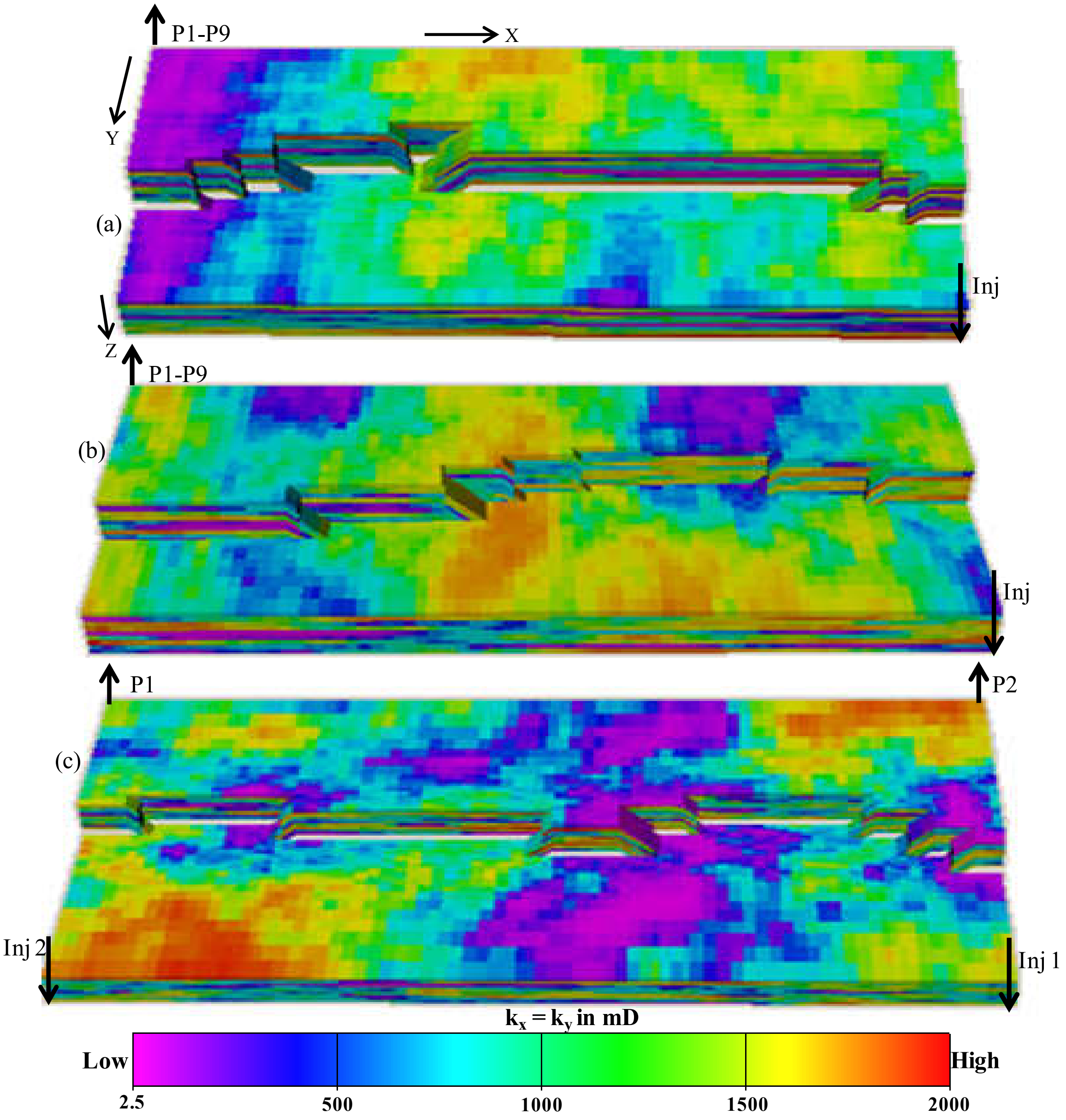

Supplementary Figure S3. Geometric configurations of truth models of three reservoir test cases showing the permeability field in horizontal directions and their well locations. (**a**) Reservoir A. (**b**) Reservoir B. (**c**) Reservoir C.

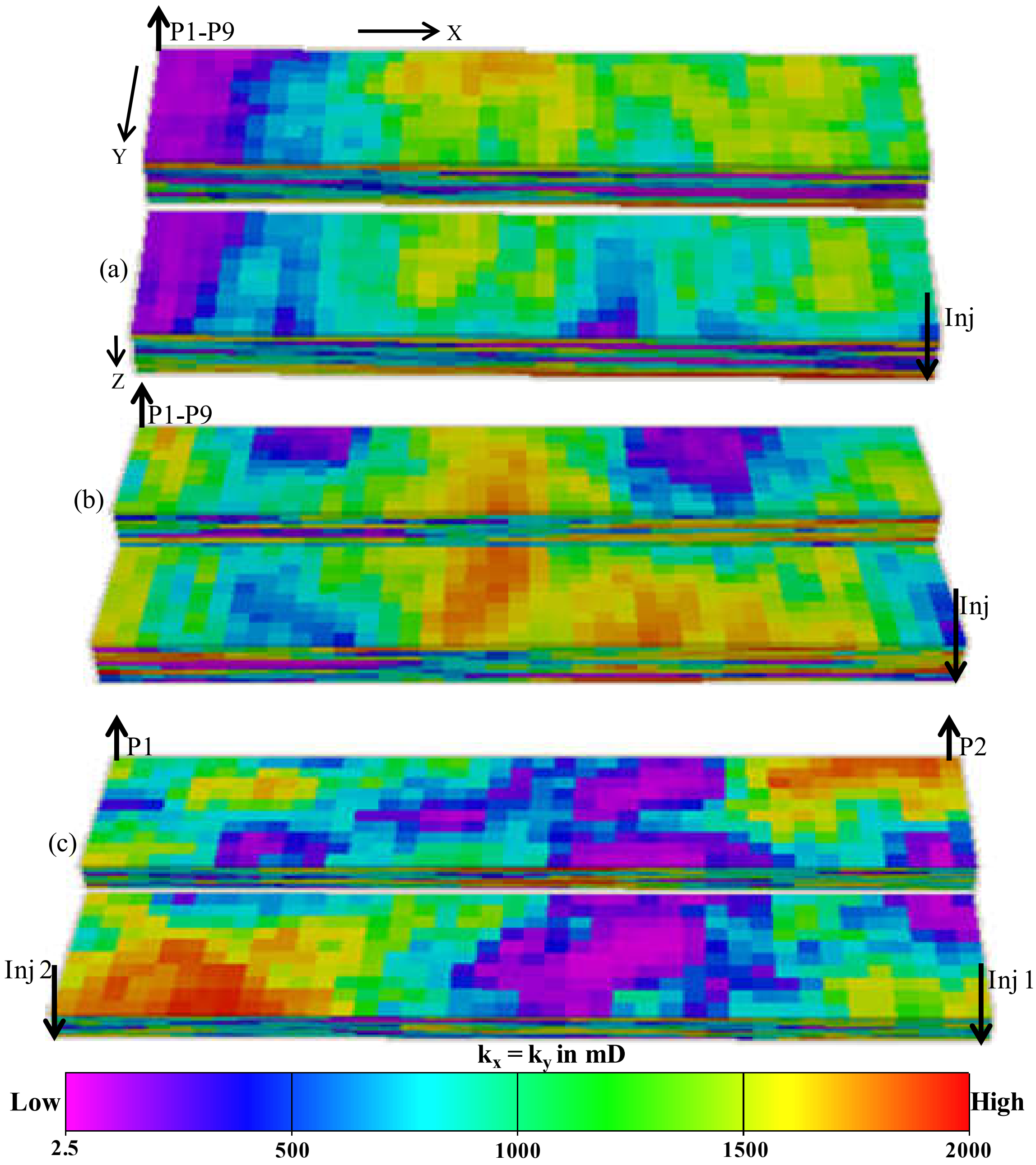

Supplementary Figure S4. Geometric configurations of upscaled model versions of three truth models (presented in Supplementary Fig. S3) showing the permeability field in horizontal directions and their well locations. (**a**) Reservoir A. (**b**) Reservoir B. (**c**) Reservoir C.

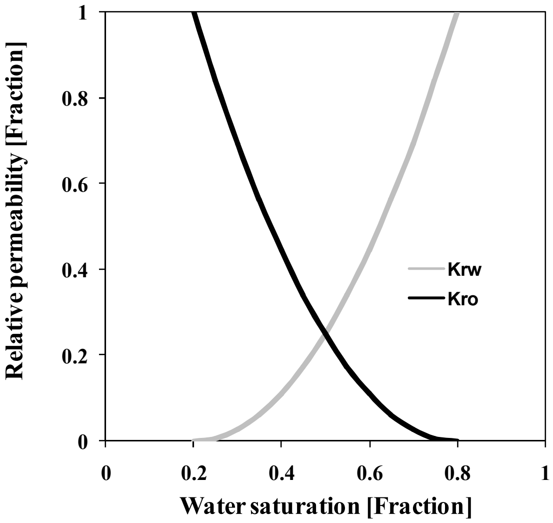

Supplementary Figure S5. Relative permeability curve used for two-phase numerical flow simulation in this study. Drawn from Christie and Blunt[46].

Supplementary Tables

Supplementary Table S1. Distribution of petrophysical properties of three geological models.

Reservoir	Permeability [mD]		Net-To-Gross		Porosity	
	Exp	Std	Exp	Std	Exp	Std
A	1000	425	0.50	0.125	0.25	0.125
B	1000	450	0.50	0.20	0.25	0.10
C	1000	500	0.60	0.20	0.25	0.0625

Supplementary Table S2. Variogram of petrophysical properties modelling of three geological models.

Reservoir	Ranges in Three Directions [m]			Azimuth Direction	Dip
	Major	Minor	Vertical		
A	4000	2000	10	0	0
B	5000	2000	10	0	0
C	500	500	2	0	0

Supplementary Table S3. PVT properties of dead oil.

Pressure, P (bara)	Formation Volume Factor, B_o (dimensionless)	Viscosity, μ_o (cp)
300	1.055	2.85
500	1.045	2.86
600	1.040	2.87
750	1.035	2.88
1000	1.010	2.89
1200	1.00	2.90

Supplementary References

44. Vergnes, C., Lallier, F., Bergey, P., Berthet, P. & Vignau, S. Stochastic Simulations of Fault Relays and Induced Fault Transmissibility at Reservoir Scale-Method and Application. Paper N110 09, Presented at the 77th EAGE Conference and Exhibition, Madrid, Spain, 1-4 June 2015, DOI: 10.3997/2214-4609.201413273.

45. Irving, A. D., Kuznetsov, D., Robert, E., Manzocchi, T. & Childs, C. Optimization of Uncertain Structural Parameters With Production and Observation Well Data. SPE Reservoir Evaluation & Engineering **17(04)**, 547-558 (2014).

46. Christie, M. A. & Blunt, M. J. Tenth SPE comparative solution project: A comparison of upscaling techniques. SPE Reservoir Evaluation & Engineering **4(04)**, 308-317 (2001).
